# Dietary inclusion of olive cake alone or in combination with *Saccharomyces cerevisiae* in Black goat kids: Implications for performance and health

**DOI:** 10.14202/vetworld.2024.2497-2505

**Published:** 2024-11-13

**Authors:** Belal S. Obeidat, Jafar Al-Khazaleh, Milton G. Thomas, Mohammed D. Obeidat, Basheer M. Nusairat

**Affiliations:** 1 Department of Animal Production, Faculty of Agriculture, Jordan University of Science and Technology, Irbid, Jordan; 2 Department of Nutrition and Food Processing, Faculty of Agricultural Technology, Al-Balqa Applied University, Al-Salt, Jordan; 3 Department of Animal Science, Texas A&M AgriLife Research, Beeville Texas, USA

**Keywords:** black goat kids, blood parameters, carcass characteristics, olive cake

## Abstract

**Background and Aim:**

To address the problems associated with the availability and prices of conventional feeds, researchers have started to include alternative feeds to reduce the cost of diets and increase profitability. This study examined the influences of olive cake (OC), either alone or in combination with *Saccharomyces cerevisiae* (SC), in the diet of black kids.

**Materials and Methods:**

Thirty kids were distributed into three treatments: A free OC diet (control [CON]), OC (20% OC), and OCSC (20% OC with 1 g SC head/d). While penned individually, the kids were fed daily and allowed to acclimate to their diets and pens for 7 days. After acclimation, the kids were fed the prescribed diets for 63 days. Five kids were chosen from each group on day 50 of the feeding period and moved to the metabolic cages to evaluate nutrient digestibility and N balance. At the end of the study, all kids were slaughtered to evaluate carcass characteristics and meat quality.

**Results:**

Feeding the OCSC diet increased (p = 0.035) DM intake for kids compared with the OC and CON. The CP intake was higher (p = 0.021) in the OCSC group than in the CON group. Growth performance parameters were higher (p ≤ 0.031) in the OCSC group than in the CON diet. Feed conversion efficiency was improved (p = 0.052) by incorporating OCSC compared with the CON diet. Consequently, economic return was enhanced (p = 0.003) by feeding the OC and OCSC groups. Kids fed the OCSC had a higher cold carcass weight (p = 0.054) than those fed the CON diet. The OCSC group also showed increased (p ≤ 0.027) loin cut and intermuscular fat weights compared with the OC and CON groups. All measured serum metabolites were similar in CON and groups incorporating OCs or OCSCs.

**Conclusion:**

The results showed that adding 20% OC combined with SC to the diet of growing kids improved some growth and carcass characteristic parameters without affecting their health status. Notably, using OC along with SC could be an option for feed cost reduction in kids’ diets.

## Introduction

Nutrition is a principal factor affecting livestock health and productivity. However, feed shortages, rangelands, and rising feed prices are among the challenging obstacles that may affect animal nutrition and force livestock managers to re-evaluate their feeding practices. Alternative feeds or appropriate feed additives are important for minimizing feeding costs, enhancing feed effectiveness, and ensuring livestock system sustainability.

Olive cake (OC) is a potential feed resource used in ruminant diets [1, 2]. It is an agro-industrial byproduct obtained after extracting olive oil. It contains husk, stone wall, pulp, kernel, and residual oil [3], constituting 35%–40% of the total processed olive weight [4, 5]. Olives are cultivated in the Mediterranean [6], where 90% of global olive oil production occurs [2]. Domestically, olive oil extraction plants produce large seasonal amounts of OC, and these by-products are not easily decomposed, posing environmental risks [7, 8]. Abo Omar [9] found that 15% OC did not influence the productivity of Awassi lambs. A recent study by Obeidat and Thomas [3] reported that including OCs of 7.5% and 15% in the diets of growing Black goat kids produced similar growth and carcass and meat quality variables. Previous studies [2–5] have concluded that replacing traditional feeds with alternative feeds such as OC could be economically viable without affecting the health and performance of animals.

OC is a high source of fiber [10], is regarded as a low-quality feedstuff, and contains anti-nutrient factors [10, 11]. Therefore, improving the nutritional value of the feed before animal feeding is needed for effective use [8]. Feed additives such as probiotics may affect the microbial population in ruminant animals, especially when provided in proper types [12, 13]. Therefore, they may be a good option for increasing the use of OC. Another purpose of using these additives is to mitigate antibiotics because they improve the performance of gut microbes and animals [14]. *Saccharomyces cerevisiae* (SC) is a well-known yeast that is used in cattle [15, 16], growing lambs [17], and ewe [18] diets. As a result, improving the activity of ruminal microbes would positively improve the breakdown of fiber [15], enhance the immune system, and, as a result, may increase the amount and quality of animal products [16, 19, 20].

Consistent with the abovementioned facts, the insertion of OCs and SCs into livestock diets may reduce the costs of animal feeding, improve livestock performance, alleviate environmental risk, and, in turn, mitigate climate change. For the current study, we hypothesized that including a proper level of OC as an alternative to replacing wheat straw alone or combined with SC may positively impact growth, economic returns, and carcass quality and attributes without detrimental effects on animal health. This study was conducted to evaluate the effects of incorporating OC alone or in combination with SC into the diet of kids on metabolic responses, health, growth, and carcass features.

## Materials and Methods

### Ethical approval

All procedures used in this experiment were approved by the Jordan University of Science and Technology Animal Care and Use Committee (#16/04/12/637).

### Study period and location

The study was conducted from August 2023 to November 2023 at the Jordan University of Science and Technology, Agricultural Research and Training Unit, Faculty of Agriculture.

### Experimental groups

Thirty male kids (initial body weight: 17.00 ± 0.58 kg; Age: 92.5 ± 2.25 days, standard deviation) were randomly allocated into three dietary groups of 10 male kids each: Control (CON) group, 20% OC, and 20% OC plus 1 g kid/d/SC (Levucell^®^SC, Lallemand, France; 3 × 10^9^/g) (OCSC). OC was obtained from a local olive oil press in Irbid, Jordan. The cost of the OC was $50 per ton. The chemical compositions of the OCs were 89.6%, 7.7, 57.8, 35.4, and 10.1% for dry matter (DM), crude protein (CP), neutral detergent fiber (NDF), acid detergent fiber (ADF), and ether extract (EE), respectively. In addition, OCs contained 2.20, 120.9, and 272.9 mg/100 g of anthocyanin, flavonoid, and phenol, respectively. Total anthocyanins, total flavonoids, and total phenols were analyzed using procedures described by Uribe *et al*. [21], Kim *et al*. [22], and Chuah *et al*. [23], respectively. Diets were 16% CP, isonitrogenous, and developed to match the NRC [24] requirements for growing kids. While penned individually (1 m × 1.5 m), kids were fed every day at 8:00 and 15:00 and allowed to acclimate to their diets and pens for 7 days. After acclimation, the kids were fed the prescribed diets for 63 days. The kids had free access to fresh water and diet throughout the feeding period. Feed that was collected and leftover was measured every day, and then 20% of it was subsampled, dried at 55°C for 48 h, processed through a 1-mm screen, and prepared for further analysis. Samples were placed in nylon bags at 20°C and kept for DM and other nutrients to be examined to determine daily nutritional intake. The weight of the kids was measured immediately before feed intake in the morning to minimize weighing variation and to estimate the average daily gain (ADG), total gain, feed conversion ratio (FCR), and (ADG; FCR = DMI ÷ weight gain) at the start of the study and every 2 weeks thereafter. Feed and refusal samples were evaluated for DM (100°C in an air-forced oven for 24 h), CP (Kjeldahl process), and EE (Soxtec method) as described by the AOAC [25] protocol. NDFs and ADF were evaluated using procedures described by Van Soest *et al*. [26] with an ANKOM^2000^ fiber analyzer (Ankom Technology Cooperation, Fairport, NY, USA).

A partial budget analysis was executed to confirm the cost–benefit analysis of adding different amounts of OC to the diets of kids. The total cost of each diet ($US/1000 kg) was calculated by summing the total cost of each component based on current prices. The costs of labor, water, and electricity were considered in the diet cost. The gain cost was obtained by multiplying FCR with the cost of diet ($US/kg diet).

### Digestion and N balance experiments

Starting from day 50 of the study, five kids were randomly selected from each group to determine nutritional digestibility and N balance and moved to individual-sized metabolic cages measuring 1.05 m × 0.80 m. Then, the kids were assigned 5 days to collect data. Daily feces from the kids were obtained, weighed, and recorded, with 10% kept for further analysis. In addition, during the same time of fecal collection, urine was collected, weighed, and stored in plastic containers, with the remaining 5% stored at –20°C to evaluate the nitrogen (N) balance (N intake, N lost in feces and urine, retained N (g/d), and N retention%. To ensure ammonia retention, each urine cup contained 50 mL of 6 N hydrogen chloride. Each fecal sample collected from each kid was combined to create a single sample for each kid. Then, the samples were exposed to an oven at 50°C to achieve a consistent dehydrated weight. As previously described by AOAC [25] and Van Soest *et al*. [26], the dried samples were ground and analyzed for DM, NDF, ADF, CP, and EE content. The collected urinary samples were combined for each kid to obtain one sample for each kid. Then, they were analyzed for N content using the Kjeldahl procedure.

### Slaughtering procedures

All kids were slaughtered at the end of the study (i.e., 63 days of the feeding period) to evaluate carcass characteristics. The kids were harvested at 9:00 am, approximately 18 h after the last feeding. The live body weight was measured before slaughter. Kids were sent after that to slaughtering areas and slaughtered by trained personnel following the halal method, and hot carcass weights were taken and recorded. Non-edible components of the carcass (lungs and trachea, heart, spleen, liver, and kidney) were obtained, weighed, and recorded after harvest. After chilling the carcasses for 24 h at 4°C, their cold weights were measured and recorded. The dressing percentage was calculated by dividing the cold carcass weight by the live fasting body weight. One day following slaughter, measurements were made of the *Longissimus dorsi* muscles and cooled corpses to determine the linear dimensions of fat depth (C), eye muscle depth (B), eye muscle width (A), tissue depth (GR), rib fat depth (J), and eye muscle area. The carcasses were then fabricated into four sections: rack, leg, shoulder, and loin cuts. Carcass cut weights (shoulder, racks, loins, and legs) were weighed and recorded. To assess the quality of the meat, *Longissimus dorsi* muscles were removed from the loin cut and vacuum-packed inside an ultra-low laboratory freezer set at –20°C for 2 weeks in plastic bags until further analysis.

### Meat quality measurements

Meat quality was measured using the following parameters: pH, shear force, cooking loss (CL), water-holding capacity (WHC), and color coordinate (CIE L*, a*, b*). The frozen *Longissimus dorsi* muscles were defrosted overnight at 4°C in a refrigerator while they were still packed. Each muscle was divided into slices of varying thickness, and each piece’s meat quality was assessed using a unique test. Slices of the hue 15 mm thick were recorded. After placing each slice on a polystyrene tray and covering it with a porous sheet, the slices were allowed to oxygenate for 2 h at 4°C. CL was computed using 25-mm-thick slices of meat. Before cooking, the slices were weighed, placed in plastic bags, and then cooked sous vide for 90 min at 75°C in a water bath. They were weighed once more following cooking. The cooked slices were kept at 4°C overnight to collect shear force data [27]. Subsequently, they were divided into six smaller samples or cores, each measuring 1 cm^3^. In addition, the maximum force (kg) required to shear cooked meat scores was ascertained using a Warner-Bratzler (WB) shear blade with a triangular slot sharp edge (GR Manufacturing Co. 1317 Collins LN, Manhattan, Kansas, 66502, USA) on a Salter Model 235. The method proposed by Grau and Hamm [28] was used to obtain the WHC. Five grams of raw meat were cut into small pieces, placed between two quartz plates and filter papers, and crushed for 5 min with a 2.5 kg weight before being removed and weighed. WHC percentage = (Initial weight – Final weight) × 100/Initial weight.

### Blood parameters

Using a plain vacutainer, 10 mL of blood samples were collected from each kid’s jugular vein at 8:00 a.m. on days 1, 30, and 60 of the experiment. At 9:00 a.m., a centrifuge was used to separate blood serum. Serum samples were stored for 14 days at –20°C until analysis. The biochemical parameters of blood, including triglycerides, creatinine, low-density lipoprotein (LDL), high-density lipoprotein (HDL), cholesterol, urea N content, and serum glucose, were examined. In addition, commercial kits (Biolabo S.A.S., Less Hautes Rivers, Maizy, France) were used to measure liver enzymes such as aspartate aminotransferase (AST), alkaline phosphatase (ALP), and alanine aminotransferase (ALT). A spectrophotometer was used for the measurements (Jenway 6105 UV/Vis, Model 6105, Janeway LTD, Felsted, Dunmow ESSEX CM6 3LB, UK).

### Statistical analysis

The data were analyzed using the MIXED procedure of SAS (version 8.1, 2000, SAS Institute Inc., Cary, NC, USA). In the statistical model, only the treatments (CON, OC, and OCSC) were considered fixed effects. The least-squares means were determined using the relevant pair-wise t-tests where the fixed effects demonstrated significance (p < 0.05).

## Results

### Nutrient intake and growth performance

As illustrated in Table-1, DM intake was higher (p = 0.035) due to the inclusion of OCSCs in the diet than in the OC and CON. The CP intake was higher (p = 0.005) in the OCSC group than in the CON group, but the OC group had a similar CP intake as the CON and OCSC groups. Moreover, kids in the OC and OCSC groups showed a higher (p < 0.0001) intake of EE than those in the CON group. However, NDF, ADF, and ME were comparable (p ≥ 0.162) among the three diet groups. Kids in the OCSC group had higher (p < 0.051) final BW, total gain, and ADG than those in the CON group, but those in the OC group had similar values compared with the CON and COSC groups. Feed conversion efficiency was improved (p = 0.052) by incorporating OCSCs into the diet compared with the CON diet. Consequently, economic return was increased (p = 0.003) in the OC and OCSC groups relative to the CON group. The body dimensions of the kids (Table-1) were similar (p ≥ 0.669) among the dietary groups.

**Table-1:** Effects of OC and SC supplementation on nutrient intake, growth performance, and body measurements in Black goat kids.

Item	Diet^a^			SEM

	CON	OC	OCSC	
Nutrient intake				
Dry matter (g/d)	847^a^	857^a^	881^b^	13.8
Neutral detergent fiber (g/d)	271	257	264	6.9
Acid detergent fiber (g/d)	119	115	118	2.1
Crude protein (g/d)	136^a^	138^ab^	142^b^	2.4
Ether extract (g/d)	8^a^	25^b^	26^b^	0.3
Metabolizable energy (Mcal/d)	1.97	1.94	1.99	0.025
Initial weight (kg)	16.8	17.1	17.1	0.58
Final weight (kg)	23.9^a^	25.1^ab^	26.3^b^	1.03
Total gain (kg)	7.1^a^	8.0^ab^	9.3^b^	0.81
Average daily gain (g/d)	105^a^	127^ab^	147^b^	15.2
Feed conversion ratio	8.0^a^	7.3^ab^	6.1^b^	0.82
Cost ($US/kg gain)	3.39^b^	2.56^a^	2.15^a^	0.314
Body measurements (cm)				
Body height	63.2	63.7	64.2	0.90
Body length	67.2	68.2	67.5	1.36
Heart girth	63.1	63.5	63.5	0.82

a For 63 days, the diets were OC-containing diet (n = 20) and control diet (CON; n = 10). Ten kids received an OC diet containing 20% DM and OC, while the other 10 received an OC diet along with 1 g/head/d of dietary DM supplemented with SC for 63 days. Within a row, means without common letters differ at p ≤ 0.05. CON=Control, OC=Olive cake, DM=Dry matter, SC=*Saccharomyces cerevisiae*, SEM=Standard error of the mean.

### Nutrient digestibility and N balance

The digestibility of DM and NDF was improved (p ≤ 0.054) in the OCSC group compared with the OC and CON groups. However, the other digestibility values (CP, ADF, and EE) were similar (p ≥ 0.135) among the three diet groups (Table-2). The amounts of N obtained and lost through feces and urine were comparable among the three diet groups (p ≥ 0.110). Moreover, the efficiency of N retained per 100 g of protein consumed did not differ among the dietary groups. However, the N balance (g/d) was higher (p = 0.046) in the OCSC group than in the CON group, but the OC diet was similar to that of the other groups.

**Table-2:** Nutrient digestibility and N balance in Black goat kids fed OC and supplemented with SC.

Item	Diet^a^			SEM

	CON	OC	OCSC	
Digestibility coefficients				
Dry matter	77.5^a^	77.3^a^	80.2^b^	1.14
Neutral detergent fiber	60.3^a^	63.2^a^	67.5^b^	2.12
Acid detergent fiber	47.9	47.4	51.9	2.18
Crude protein	78.1	76.9	78.0	1.64
Ether extract	71.4	77.3	78.0	3.33
N balance				
N intake (g/d)	24.7	24.9	26.6	0.89
N lost in urine (g/d)	7.1	6.5	7.0	0.34
N lost in feces (g/d)	6.7	7.2	6.4	0.41
N retained (g/d)	10.9^a^	11.3^ab^	13.2^b^	1.00
Retention (g/100 g)	43.9	44.8	49.7	2.71

a For 63 days, the diets were OC-containing diet (n = 20) and control diet (CON; n = 10). Ten kids received an OC diet containing 20% DM and OC, while the other 10 received an OC diet along with 1 g/head/d of dietary DM supplemented with SC for 63 days. Within a row, means without common letters differ at p ≤ 0.05. CON=Control, OC=Olive cake, DM=Dry matter, SC=*Saccharomyces cerevisiae*, SEM=Standard error of the mean, N=Nitrogen.

### Carcass and non-carcass components

The effects of OC and SC added to the diets of Black goats on carcass characteristics and loin tissues are presented in Table-3. Cold carcass weight was higher (p = 0.054) in the OCSC group than in the CON group, but the OC diet was similar to both groups. However, the incorporation of OCs and SCs did not affect (p ≥ 0.185) other measures, such as fasting live weight, hot carcass weight, dressing percentage, and non-carcass components. In addition, no notable differences were observed in carcass cut weights of the shoulder, racks, loins, and legs. The inclusion of OCSC increased (p ≤ 0.027) loin cut weight and intermuscular fat compared with the OC and CON groups (Table-3). However, the total lean muscle percentage was higher (p = 0.014) in the OC and OCSC groups than in the CON group.

**Table-3:** Carcass characteristics and loin tissues of Black goat kids fed OC and supplemented with SC.

Item	Diet^a^			SEM

	CON	OC	OCSC	
Fasting live weight (kg)	23.2	24.1	25.3	1.18
Cold carcass weight (kg)	10.4^a^	10.8^ab^	11.8^b^	0.58
Hot carcass weight (kg)	11.4	12.0	12.5	0.60
Dressing percentage	45.0	45.0	46.6	1.13
Carcass cut weight (kg)	9.7	10.0	10.8	0.76
Non-carcass components (kg)	1.3	1.3	1.3	0.06
Loin weight (g)	338.0^a^	364.4^a^	455.8^b^	28.6
Subcutaneous fat (g)	19.6	18.0	23.1	2.56
Intermuscular fat (g)	17.1^a^	14.3^a^	23.8^b^	3.30
Total lean (g/100 g)	43.7^a^	47.8^b^	49.2^b^	1.72
Total fat (g/100 g)	10.8	8.9	10.3	1.07
Total bone (g/100 g)	34.6	32.9	29.6	3.19
Meat: fat ratio	4.5	5.7	5.1	0.73
Meat to bone ratio	1.4	1.5	1.7	0.20

a For 63 days, the diets were OC-containing diet (n = 20) and control diet (CON; n = 10). Ten kids received an OC diet containing 20% DM and OC, while the other 10 received an OC diet along with 1 g/head/d of dietary DM supplemented with SC for 63 days. Within a row, means without common letters differ at p ≤ 0.05. CON=Control, OC=Olive cake, DM=Dry matter, SC=*Saccharomyces cerevisiae*, SEM=Standard error of the mean.

With the exception of rib fat depth, measurements of the other carcass linear dimensions did not differ among the treatments (Table-4). Rib fat depth was higher (p = 0.052) in the OCSC group than in the CON group, whereas the rib fat depth in the OC group was similar to that in the CON and OCSC groups.

**Table-4:** Linear dimensions of the *Longissimus dorsi* muscle of Black goat kids fed OC and supplemented with SC.

Item	Diet^a^			

	CON	OC	OCSC	SEM
Eye muscle width (A) (mm)	41.51	44.61	43.22	1.611
Eye muscle depth (B) (mm)	17.83	19.67	19.60	1.158
Fat depth (C) (mm)	1.00	1.00	1.05	0.041
Tissue depth (GR) (mm)	7.34	9.67	9.59	1.20
Rib fat depth (J) (mm)	1.23^a^	1.67^ab^	1.74^b^	0.222
Leg fat depth (L3) (mm)	1.23	1.22	1.34	0.212
Shoulder fat depth (S2) (mm)	1.00	1.00	1.06	0.040
Eye muscle area (cm^2^)	6.62	7.34	7.08	0.339

a For 63 days, the diets were OC-containing diet (n = 20) and control diet (CON; n = 10). Ten kids received an OC diet containing 20% DM and OC, while the other 10 received an OC diet along with 1 g/head/d of dietary DM supplemented with SC for 63 days. Within a row, means without common letters differ at p ≤ 0.05. CON=Control, OC=Olive cake, DM=Dry matter, SC=*Saccharomyces cerevisiae*, SEM=Standard error of the mean.

### Meat quality characteristics

Regarding the different characteristics of the *Longissimus dorsi* muscle in Black goat kids, no changes (p > 0.101) were found among the treatments (Table-5).

**Table-5:** Physicochemical properties of the *Longissimus dorsi* muscle of Black goat kids fed OC and supplemented with SC.

Item	Diet^a^			

	CON	OC	OCSC	SEM
pH	5.90	5.89	5.88	0.017
Color co-ordinates^b^				
L* (whiteness)	36.19	37.22	36.32	1.156
a* (redness)	2.21	2.12	1.81	0.402
b* (yellowness)	23.28	22.61	21.41	1.36
Shear force (kg/cm^2^)	6.12	7.31	7.00	0.540
Water-holding capacity (%)	39.71	38.62	36.93	1.524
Cooking loss (%)	45.90	45.90	44.24	1.142

a For 63 days, the diets were OC-containing diet (n = 20) and control diet (CON; n = 10). Ten kids received an OC diet containing 20% DM and OC, while the other 10 received an OC diet along with 1 g/head/d of dietary DM supplemented with SC for 63 days. Within a row, means without common letters differ at p ≤ 0.05. CON=Control, OC=Olive cake, DM=Dry matter, SC=*Saccharomyces cerevisiae*, SEM=Standard error of the mean.

b L* indicates lightness/darkness co-ordinate, a* is the red/green co-ordinate, and b* is the yellow/blue co-ordinate.

### Blood parameters

All measured metabolites and liver enzymes present in blood serum were not affected (p ≥ 0.088) by incorporating OC or OCSC into the diet (Table-6).

**Table-6:** Effects of OC and SC supplementation on liver enzymes and blood metabolites in Black goat kids.

Item	Diet^a^			SEM

	CON	OC	OCSC	
Liver enzyme levels (IU/L)				
Alkaline phosphatase	77.7	96.2	85.0	10.49
Aspartate aminotransferase	35.6	29.6	27.1	3.95
Alanine aminotransferase	14.3	10.7	11.0	2.82
Blood metabolites (mg/dL)				
Blood urea nitrogen	23.6	27.4	25.3	1.90
Glucose	66.0	61.7	65.1	2.32
Cholesterol	63.1	67.6	68.9	4.03
High-density lipoprotein	38.2	41.6	41.7	2.53
Low-density lipoprotein	21.6	22.4	24.2	2.62
Triglycerides	16.0	16.6	14.9	1.34
Creatinine	0.90	1.03	0.98	0.078

a For 63 days, the diets were OC-containing diet (n = 20) and control diet (CON; n = 10). Ten kids received an OC diet containing 20% DM and OC, while the other 10 received an OC diet along with 1 g/head/d of dietary DM supplemented with SC for 63 days. Within a row, means without common letters differ at p ≤ 0.05. CON=Control, OC=Olive cake, DM=Dry matter, SC=*Saccharomyces cerevisiae*, SEM=Standard error of the mean.

## Discussion

In this study, an assessment of the ingredients and chemical composition of the diets (Table-7) [24] indicated that only EE content differed significantly between the CON and OC groups due to the replacement of wheat straw in the CON group with OC. According to Chiofalo *et al*. [29], OC contains more EE because of its residual oil concentration. Moreover, García-Rodríguez *et al*. [7] reported greater EE content in a diet containing OCs than in a CON group. Variations in the nutrient content of OCs may be influenced by many factors, including season of the year, variety, processing type, ripening stage of olives, and (or) geographical region [7, 17, 30]. Consequently, OC must be examined before its incorporation into diets to verify its nutritional value.

**Table-7:** Components and nutrient content of diets offered to Black goat kids.

Item	Diet^a^	

	CON	OC
Ingredients (% DM)		
OC	0	20.0
Wheat straw	27.0	7.0
Soybean meal	21.0	18.5
Barley grain	50.0	52.5
Limestone	0.9	0.9
Vitamin–mineral premix	0.1	0.1
Salt	1.0	1.0
Cost (US$/1,000 kg)^b^	422	353
Nutrients		
DM, %	92.5	91.2
Neutral detergent fiber, % DM	31.9	30.0
Acid detergent fiber, % DM	13.9	13.4
Crude protein, % DM	16.0	16.1
Ether extract, % DM	0.96	2.97
Metabolizable energy (Mcal/kg)^c^	2.32	2.26

a For 63 days, the diets were OC-containing diet (n = 20) and control diet (CON; n = 10). Ten kids received an OC diet containing 20% DM and OC, while the other 10 received an OC diet along with 1 g/head/d of dietary DM supplemented with SC for 63 days. b Based on cost of components in 2023. c Based on the NRC tabular value, NRC [24]. DM=Dry matter, CON=Control, OC=Olive cake, SC=*Saccharomyces cerevisiae.*

The OC and OCSC diets are less expensive than the CON diet. This cost reduction can be explained by substituting the wheat straw portion with OCs (Table-7) [24]. These findings parallel previous studies by Obeidat [17], Bionda *et al*. [31], and El Otmani *et al*. [32], showing that using unconventional feeds contributed to decreasing the cost of animal feeding.

Only DM, CP, and EE intake in this study differed among the groups. The highest DM intake in the OCSC group could be ascribed to the presence of SC-enhanced ruminal bacterial viability. However, EE intake was higher in the OC and OCSC groups than in the CON group due to the greater EE levels observed in the OC and OCSC groups (Table-7) [24]. Similarly, Obeidat [17] found that EE intake was more significant in a diet containing 15% OC than in a CON diet in Awassi lambs. However, in that study of Obeidat [17], no differences were observed in other nutrient intake among the CON, OC, and combination of OC and SC at 0.5 g/head/day.

Moreover, a study by Vargas-Bello-Pérez *et al*. [33] reported that sheep feed intake was not affected by the inclusion of 10% or 25% OC in the diet. Furthermore, Cabiddu *et al*. [34] reported a decrease in feed intake when OC was added to the diet of lactating cows. Although the majority of studies have agreed regarding the effect of feeding livestock diets containing OC, differences in feed consumption, if any, between studies are due to the geographical origin of olives, level of OC inclusion, season of the year, extraction method, and ripeness stage of the fruit.

The results presented here are in line with Moallem *et al*. [35] on the influence of SC on feed intake. This study indicated that yeast supplementation increased DM intake by 2.5% in dairy cows during the hot season. Furthermore, AlZahal *et al*. [36] found that DM intake improved in dairy cows fed diets supplemented with active dry SC. On the other hand, Ben Saïd *et al*. [37] and Soren *et al*. [38] found that DM intake did not differ between sheep fed a SC-supplemented diet. In this study, when comparing the OCSC and CON diets, there was a discernible improvement in feed conversion efficiency.

In this study, the kids’ final BW, total gain, and ADG were improved in the OCSC group compared with the CON group. The higher improvement in kids’ performance can be attributed to their higher DM, CP, and EE intake levels in the OCSC. However, a previous study by Obeidat [17] on Awassi lambs showed no significant differences in TWG and ADG among diets incorporated with 15% OC only or with SC. According to a study by Beken and Sahin [39], ultimate BW and total gain in Awassi lambs fed a diet containing 20% OC combined with concentrate did not differ from those in lambs fed a CON diet. However, Mioc *et al*. [40] found that feeding Pramenka lambs a 30% OC diet, as opposed to those fed a 15% OC or CON diet, led to lower ultimate BW and ADG.

Moreover, Awassi lambs fed OC-containing diets grew more quickly, had higher final BW, and gained more overall than lambs fed the CON diet [41]. Mousa *et al*. [42] similarly observed that daily increases and lamb weaning weights were more significant in the yeast-supplemented groups than in the CON group, similar to our findings. Our results, however, contradict those of Obeidat [17], Soren *et al*. [38], and Zaleska *et al*. [43], who reported no difference in the BW and ADG of lambs after SC supplementation. Although the DM intake, final BW, TWG, and ADG values were more significant in the OCSC group than in the CON group, the kids’ linear body measurements were similar among the diet treatments. Therefore, these measurements cannot be appropriately used to predict animal performance.

Moreover, economic outcomes were enhanced in the OC and OCSC groups compared with the CON group (Table-1). This may have occurred because unconventional feeds are more cost-effective than alternative feeds [3]. Using OCs reduces the cost of gain in kids on OC diets (Table-1) and diet expenses (Table-7) [24]. Surprisingly, a previous study by Obeidat [17] on Awassi lambs found no differences in feed efficiency among diets containing 15% OC alone or SC.

In the present study (Table-2), only DM and NDF digestibility was improved by the OCSC diet compared with the other diets. The other digestibility values (CP, ADF, and EE) were similar among the three groups. This result is consistent with the report of Ferraretto *et al*. [44], who observed that supplementing a high-starch diet with varying doses of live SC improved DM and NDF digestibility in the rumen of lactating cows. However, a study by Soren *et al*. [38] suggested that adding yeast to the diet increased the ability of sheep to digest ADF but did not affect DM, CP, or NDF digestibility. Furthermore, Mousa *et al*. [42] reported that adding 5 or 7.5 g/h/d of live dried yeast to the diet of Rahmani ewes improved the digestibility of DM and CP. In addition, a study by Ben Saïd *et al*. [37] found that while SC supplementation increased the digestibility of DM, it did not affect the digestibility of CP.

Our findings are consistent with those of Awawdeh and Obeidat [41], who indicated no differences in the digestibility of DM, CP, and ADF when OC was included in the diets of Awassi lambs. Yáñez-Ruiz and Molina-Alcaide [2] reported that adding two stages of OC increased the level of condensed tannins in the diet, which reduced the digestibility of DM, OM, NDF, ADF, and CP. In addition, Martín García *et al*. [45] reported low *in vitro* digestibility values for olive leaves and OC, particularly for CP digestibility.

In the current study, the N balance parameters were similar among the groups, except for the amount of retained N per day, which was higher in the OCSC group than in the CON group (Table-2). Obeidat [18] showed that the addition of low or high SC levels enhanced the N balance parameters of nursing Awassi ewes. However, in Obeidat’s study [17], all N balance parameters were similar among diets (CON vs. OC vs. OCSC) among Awassi lambs. Furthermore, Ismail and Obeidat [46] found that the addition of olive leaves to the diet of Awassi lamb did not alter N balance parameters. Aljamal *et al*. [47] also observed this result when compared with ewes fed a CON diet, nursing Awassi ewes given OC at a rate of 25% did not exhibit any change in N balance parameters. Previous studies [37, 38] using sheep-fed diets supplemented with yeast found no changes in N intake, output, or retention.

In terms of carcass characteristics and loin tissues (Table-3), dietary OCSC supplementation had only a significant impact on cold carcass weight, loin weight, and intermuscular fat weight. Moreover, dietary OC and OCSC supplementation increased the total lean percentage. The changes in these variables can be explained by the high final kid weights associated with the effect of SC inclusion in the diet (Table-1). The carcass attributes findings of this investigation are consistent with those of a prior study conducted by Ozdogan *et al*. [48], which observed no differences in carcass weight or yield between lambs fed diets containing 12.5% and 25% OC. Furthermore, Mioc *et al*. [40] reported that dressing percentage and carcass weight were unaffected by adding 15% OC to the diet. However, the dressing percentage and carcass weight decreased when the OC content was increased to 30%. A prior study by Hamdi *et al*. [49] found that adding OC at a rate of 280 g/d had no discernible effect on the carcass or meat quality of Barbarine lambs.

Furthermore, Awawdeh and Obeidat [41] found that OCs did not affect hot and cold carcass weight or dressing percentage. However, carcass features and intramuscular fat content improved when OC was included at 7.5% and 15% in beef cattle diets [50]. Except for a higher rib fat depth in the OCSC group than in the CON group, the other carcass linear dimensions and *Longissimus dorsi* muscle were similar among the three groups. These findings are in agreement with Issakowicz *et al*. [51], who found that yeast supplementation improved carcass weight and length in lambs fed a yeast diet.

Our findings were consistent with those of Ozdogan *et al*. [48], who did not find differences in meat quality parameters such as color coordinates, WHC, CL, shear force, or pH did not differ among the treatments in lambs fed OC at 12.5% and 25% OC. In a study conducted by Chiofalo *et al*. [50], it was found that including a high level of OC (15.0%) in the diet of beef cattle decreased shear force and CL without altering water volume compared with low levels of OC (7.5%) in CON diets.

Biochemical serum metabolites were examined as indicators of health in kids. In the current study, the diet groups did not alter all plasma biochemical indices (urea N, glucose, cholesterol, LDL, HDL, triglycerides, creatinine, AST, ALP, and ALT) (Table-1). These findings suggest that the addition of OC alone or in combination with SC did not expose the liver or the health of the animals to harmful effects. Awawdeh *et al*. [52] reported that adding up to 50% of alternative feedstuffs to the diet did not negatively affect lambs’ hematological and biochemical parameters. A previous study by Obeidat [17] on Awassi lambs showed no differences in the serum concentrations of alkaline phosphatase, aspartate aminotransferase, urea-nitrogen, or alanine aminotransferase among groups incorporated with 15% OC only or with SC compared with the CON group.

## Conclusion

This study revealed that integrating 20% OC combined with 1 g/kid/d SC has some advantages for feed intake, digestibility, growth performance, carcass quality, and meat quality in Black goat kids without showing any detrimental effects on health. Importantly, the use of OC alone or in combination with SC in this study improved the economic return by reducing feed costs. Further studies are needed at higher levels of OC inclusion in the diets of black goats and other small ruminants.

## References

[1] Abd El Tawab A.M, Shaaban M.M, Hadhoud F.I, Ebeid H.M, Khattab M.S.A (2018). Improving utilization of olive cake silage by treating with fibrolytic enzymes on digestibility and gas production in the rumen. Egypt. J. Nutr. Feeds.

[2] Yáñez-Ruiz D.R, Molina-Alcaide E (2007). A comparative study of the effect of two-stage olive cake added to alfalfa on digestion and nitrogen losses in sheep and goats. Animal.

[3] Obeidat B.S, Thomas G.M (2024). Growth performance, blood metabolites and carcass characteristics of Black goat kids fed diets containing olive cake. Animals.

[4] Dal Bosco A, Castellini C, Cardinali R, Mourvaki E, Moscati L, Battistacci L, Servili M, Taticchi A (2007). Olive cake dietary supplementation in rabbit:Immune and oxidative status. Italian J. Anim. Sci.

[5] Elbaz A.M, Farrag B, Mesalam N.M, Basuony H.A, Badran A.M.M, Abdel-Moneim A.E (2023). Growth performance, digestive function, thyroid activity, and immunity of growing rabbits fed olive cake with or without *Saccharomyces cerevisiae* or citric acid. Trop. Anim. Health Prod.

[6] Berbel J, Posadillo A (2018). Review and analysis of alternatives for the valorization of agroindustrial olive oil by-products. Sustainability.

[7] García-Rodríguez J, Mateos I, Saro C, González J.S, Carro M.D, Ranilla M.J (2020). Replacing forage by crude olive cake in a dairy sheep diet:Effects on ruminal fermentation and microbial populations in rustic fermenters. Animals (Basel).

[8] Hadhoud F.I, Kholif A.E, Abd El Tawab A.M, Shaaban M.M, Mostafa M.M.M, Ebeid H.M, Matloup O.H (2021). Partial replacement of concentrate with olive cake in different forms in the diet of lactating Barki ewes affects the lactational performance and feed utilization. Ann. Anim. Sci.

[9] Abo Omar J.M, Daya R, Ghaleb A (2012). Effects of different forms of olive cake on the performance and carcass quality of Awassi lambs. Anim. Feed Sci. Technol.

[10] Tzamaloukas O, Neofytou M.C, Simitzis P.E (2021). Application of olive by-products in livestock with emphasis on small ruminants:Implications on rumen function, growth performance, milk and meat quality. Animals (Basel).

[11] Patra A.K, Min B.R, Saxena J, Patra A.K (2012). Dietary tannins on microbial ecology of the gastrointestinal tract in ruminants. Dietary Phytochemicals and Microbes.

[12] Baker L.M, Kraft J, Karnezos T.P, Greenwood S.L (2022). The effects of dietary yeast and yeast-derived extracts on rumen microbiota and their function. Anim. Feed Sci. Technol.

[13] Hassan M.A.S, Karsli M.A (2022). The Effects of some feed additives in nutrition of ruminant animals. Int. J. Vet. Anim. Res.

[14] Elghandour M.M, Abu Hafsa S.H, Cone J.W, Salem A.Z, Anele U.Y, Alcala-Canto Y (2022). Prospect of yeast probiotic inclusion enhances livestock feeds utilization and performance:An overview. Biomass Convers. Bior.

[15] Fonty G, Chaucheyras-Durand F (2006). Effects and modes of action of live yeasts in the rumen. Biologia.

[16] Pang Y, Zhang H, Wen H, Wan H, Wu H, Chen Y, Li S, Zhang L, Sun X, Li B, Liu X (2022). Yeast probiotic and yeast products in enhancing livestock feeds utilization and performance:An overview. J. Fungi (Basel).

[17] Obeidat B.S (2017). The effects of feeding olive cake and Saccharomyces cerevisiae supplementation on performance, nutrient digestibility, and blood metabolites of Awassi lambs. Anim. Feed Sci. Technol.

[18] Obeidat B.S (2023). Effect of *Saccharomyces cerevisiae* supplementation during the suckling period on the performance of Awassi ewes. Trop. Anim. Health Prod.

[19] Bin D, Shuang X, Lu L, Xi C (2019). Research progress on application of *Saccharomyces cerevisiae* in animal production. Feed Res.

[20] Sun X, Wang Y, Wang E, Zhang S, Wang Q, Zhang Y, Wang Y, Cao Z, Yang H, Wang W, Li S (2021). Effects of *Saccharomyces cerevisiae* culture on ruminal fermentation, blood metabolism, and performance of high-yield dairy cows. Animals (Basel).

[21] Uribe E, Lemus-Mondaca R, Pasten A, Astudillo S, Vega-Gálvez A, Puente-Díaz L, Di Scala K (2014). Dehydrated olive-waste cake as a source of high value-added bioproduct:Drying kinetics, physicochemical properties, and bioactive compounds. Chilean J. Agric. Res.

[22] Kim D.O, Chun O.K, Kim Y.J, Moon H.Y, Lee C.Y (2003). Quantification of polyphenolic and their antioxidant capacity in fresh plums. J. Agric. Food Chem.

[23] Chuah A.M, Lee Y.C, Yamaguchi T, Takamura H, Yin L.J, Matoba T (2008). Effect of cooking on the antioxidant properties of colored peppers. Food Chem.

[24] NRC (2007). Nutrient Requirements of Small Ruminants:Sheep, Goat, Cervide, and New World Camelids.

[25] AOAC (1995). Official Methods of Analysis.

[26] Van Soest P.V, Robertson J.B, Lewis B.A (1991). Methods for dietary fiber, neutral detergent fiber, and nonstarch polysaccharides in relation to animal nutrition. J. Dairy Sci.

[27] Wheeler T.L, Shackelford S.D, Koohmaraie M (1996). Sampling, cooking, and coring effects on Warner-Bratzler shear force values in beef. J. Anim. Sci.

[28] Grau R, Hamm R (1953). A simple method for the determination of water binding in muscles. Naturwissenschaften.

[29] Chiofalo B, Liotta L, Zumbo A, Chiofalo V (2004). Administration of olive cake for ewe feeding:Effect on milk yield and composition. Small Rumin. Res.

[30] Marcos C.N, García-Rebollar P, de Blas C, Carro M.D (2019). Variability in the chemical composition and *in vitro* ruminal fermentation of olive cake by-products. Animals (Basel).

[31] Bionda A, Lopreiato V, Crepaldi P, Chiofalo V, Fazio E, Oteri M, Amato A, Liotta L (2022). Diet supplemented with olive cake as a model of circular economy:Metabolic and endocrine responses of beef cattle. Front. Sustain. Food Syst.

[32] El Otmani S, Chebli Y, Taminiau B, Chentouf M, Hornick J.L, Cabaraux J (2021). Effect of olive cake and cactus cladodes incorporation in goat kids'diet on the rumen microbial community profile and meat fatty acid composition. Biology (Basel).

[33] Vargas-Bello-Pérez E, Vera R.R, Aguilar C, Lira R, Peña I, Fernández J (2013). Feeding olive cake to ewes improves fatty acid profile of milk and cheese. Anim. Feed Sci. Technol.

[34] Cabiddu A, Canu M, Decandia M, Pompei R, Molle G, Ben Salem H, Nefzaoui A, Morand-Fehr P (2004). The intake and performance of dairy ewes fed with different levels of olive cake silage in late pregnancy and suckling periods. Nutrition and Feeding Strategies of Sheep and Goats Under Harsh Climates.

[35] Moallem U, Lehrer H, Livshitz L, Zachut M, Yakoby S (2009). The effects of live yeast supplementation to dairy cows during the hot season on production, feed efficiency, and digestibility. J. Dairy Sci.

[36] AlZahal O, Dionissopoulo L, Laarman A.H, McBride N.W, McBride B.W (2014). Active dry *Saccharomyces cerevisiae* can alleviate the effect of subacute ruminal acidosis in lactating dairy cows. J. Dairy Sci.

[37] Ben Saïd S, Jabri J, Amiri S, Aroua M, Najjar A, Khaldi S, Maalaoui Z, Kammoun M, Mahouachi M (2022). Effect of *Saccharomyces cerevisiae* supplementation on reproductive performance and ruminal digestibility of Queue Fine de l'Ouest adult rams fed a wheat straw-based diet. Agriculture.

[38] Soren N.M, Tripathi M.K, Bhatt R.S, Karim S.A (2013). Effect of yeast supplementation on the growth performance of Malpura lambs. Trop. Anim. Health Prod.

[39] Beken Y, Sahin A (2011). The effect of prina (olive cake) feeding methods on growth performance and behavior of Awassi lambs. Int. J. Agric. Biol.

[40] Mioc B, Pavic V, Vnucec I, Prpic Z, Kostelic A, Susic V (2008). Effect of olive cake on daily gain, carcass characteristics, and chemical composition of lamb meat. Czech J. Anim. Sci.

[41] Awawdeh M.S, Obeidat B.S (2013). Treated olive cake as a non-forage fiber source for growing Awassi lambs:Effects on nutrient intake, rumen and urine pH, performance, and carcass yield. Asian-Australas. J. Anim. Sci.

[42] Mousa K.M, El-Malky O.M, Komonna O.F, Rashwan S.E (2012). Effect of some yeast and minerals on the productive and reproductive performance in ruminants. J. Amer. Sci.

[43] Zaleska B, Milewski S, Zabek K (2015). Impact of *Saccharomyces cerevisiae* supplementation on reproductive performance, milk yield in ewes and offspring. Arch. Anim. Breed.

[44] Ferraretto L.F, Shaver R.D, Bertics S.J (2012). Effect of dietary supplementation with live-cell yeast at two dosages on lactation performance, ruminal fermentation, and total-tract nutrient digestibility in dairy cows. J. Dairy Sci.

[45] Mart??n Garc??a A.I, Moumen A, Yáñez Ruiz D.R, Molina Alcaide E (2003). Chemical composition and nutrient availability for goats and sheep of two-stage olive cake and olive leaves. Anim. Feed Sci. Technol.

[46] Ismail N, Obeidat B.S (2023). Olive leaves as alternative feed for finishing lambs:Evaluation of feed intake, nutrients digestibility, growth performance, and carcass quality. Italian J. Anim. Sci.

[47] Aljamal A.E, Obeidat B.S, Obeidat M.D (2021). Lactation performance of Awassi ewes fed diets containing either *Atriplex halimus* L. or olive cake. Italian J. Anim. Sci.

[48] Ozdogan M, Ustundag A, Yarali E (2017). Effect of mixed feeds containing different levels of olive cake on fattening performance, carcass, meat quality and fatty acids of lambs. Trop. Anim. Health Prod.

[49] Hamdi H, Majdoub-Mathlouthi L, Picard B, Listrat A, Durand D, Znaïdi I.A, Kraiem K (2016). Carcass traits, contractile muscle properties, and meat quality of grazing and feedlot Barbarian lamb receiving or not olive cake. Small Rumin. Res.

[50] Chiofalo V, Liotta L, Presti V.L, Gresta F, Di Rosa A.R, Chiofalo B (2020). Performance, carcass characteristics, and meat quality of beef cattle. Animals (Basel).

[51] Issakowicz J, Bueno M.S, Sampaio A.C.K, Duarte K.M.R (2013). Effect of concentrate level and live yeast (*Saccharomyces cerevisiae*) supplementation on Texel lamb performance and carcass characteristics. Livest. Sci.

[52] Awawdeh M.S, Dager H.K, Obeidat B.S (2020). Dietary inclusion of alternative feedstuffs had no negative effects on hematological and biochemical parameters of growing Awassi lambs. Trop. Anim. Health Prod.

